# Parkinson's Disease Modification Through Abl Kinase Inhibition: An Opportunity

**DOI:** 10.1002/mds.28858

**Published:** 2021-11-23

**Authors:** Milton H. Werner, C. Warren Olanow

**Affiliations:** ^1^ Inhibikase Therapeutics, Inc. Atlanta Georgia USA; ^2^ Department of Neurology and Department of Neuroscience Mount Sinai School of Medicine New York New York USA; ^3^ Clintrex Research Corporation Sarasota Florida USA

**Keywords:** Parkinson's disease, Abelson tyrosine kinase, disease‐modification

## Abstract

Parkinson's disease (PD) is the second most prevalent neurodegenerative disease of the central nervous system, with an estimated 5 000 000 cases worldwide. Historically characterized by the progressive loss of dopaminergic neurons in the substantia nigra pars compacta, PD pathology is now known to be widespread and to affect serotonin, cholinergic and norepinephrine neurons as well as nerve cells in the olfactory system, cerebral hemisphere, brain stem, spinal cord, and peripheral autonomic nervous system. PD pathology is characterized by the accumulation of misfolded α‐synuclein, which is thought to play a critical role in the etiopathogenesis of the disease. Animal models of PD suggest that activation of the Abelson tyrosine kinase (c‐Abl) plays an essential role in the initiation and progression of α‐synuclein pathology and neurodegeneration. These studies demonstrate that internalization of misfolded α‐synuclein activates c‐Abl, which phosphorylates α‐synuclein and promotes α‐synuclein pathology within the affected neurons. Additionally, c‐Abl inactivates parkin, disrupting mitochondrial quality control and biogenesis, promoting neurodegeneration. Post‐mortem studies of PD patients demonstrate increased levels of tyrosine phosphorylated α‐synuclein, consistent with the activation of c‐Abl in human disease. Although the c‐Abl inhibitor nilotinib failed to demonstrate clinical benefit in two double‐blind trials, novel c‐Abl inhibitors have been developed that accumulate in the brain and may inhibit c‐Abl at saturating levels. These novel inhibitors have demonstrated benefits in animal models of PD and have now entered clinical development. Here, we review the role of c‐Abl in the neurodegenerative disease process and consider the translational potential of c‐Abl inhibitors from model studies to disease‐modifying therapies for Parkinson's disease. © 2021 Inhibikase Therapeutics, Inc. *Movement Disorders* published by Wiley Periodicals LLC on behalf of International Parkinson Movement Disorder Society.

## Graphical abstract

The emergence of misfolded α‐synuclein in the substantia nigra as a result of oxidative/nitrosative stress, protein mutation, impaired clearance, and/or genetic factors leads to internalization of misfolded α‐synuclein, which activates the non‐receptor Abelson tyrosine kinase (c‐Abl), a key event in the initiation and progression of neurodegeneration in Parkinson's disease. c‐Abl activation both inactivates the ubiquitin E3 ligase parkin and creates the pathological form of α‐synuclein on the inside of the affected neurons, both events following specific tyrosine phosphorylation by c‐Abl. Treatment with a c‐Abl inhibitor reverses these processes, reactivating parkin and driving clearance of misfolded and pathological α‐synuclein through lysosomal and/or proteasomal processes. In model systems, these events result in substantial functional recovery in the brain and gastrointestinal (GI) tract. Although initial clinical trials of c‐Abl inhibitors failed, novel c‐Abl inhibitors thought to accumulate in the brain at concentrations sufficient to block c‐Abl are now being tested in the clinic and offer the opportunity to evaluate the potential of c‐Abl inhibition as a disease‐modifying therapy for patients with PD.

January Infographic : Parkinson's Disease Modification through Abl Kinase Inhibition: An Opportunity

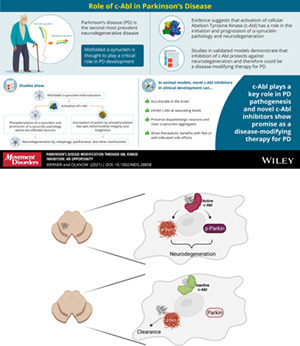

Parkinson's disease (PD) is the second most prevalent neurodegenerative disorder with an estimated 5 000 000 cases worldwide and projected doubling over the next 30 years.^1^ PD is an inexorably progressive disorder, characterized clinically by bradykinesia, rigidity, rest tremor, and gait disturbance with postural instability.^2^ The classic pathologic features of the disease include degeneration of dopaminergic (DA) neurons in the substantia nigra pars compacta (SNc) with a corresponding reduction in striatal dopamine and the accumulation of protein aggregates in cell bodies and terminals (Lewy bodies [LBs] and Lewy neurites [LN] collectively known as Lewy pathology).^3^ It is now appreciated that PD pathology is more widespread and affects non‐DA neurons including serotonin, cholinergic and norepinephrine neurons as well as nerve cells in the olfactory system, cerebral hemisphere, brain stem, spinal cord, and peripheral autonomic nervous system. Current therapies are primarily based on a dopamine replacement strategy and effectively treat the motor features of PD, particularly in the early stages of the disease.^2^ However, chronic levodopa therapy is associated with the development of motor complications in the majority of patients, and patients eventually develop intolerable disability due to features such as falling, freezing, dysphagia, neuropsychiatric disorders, autonomic dysfunction, sensory problems, and cognitive impairment with dementia that are related to the non‐DA pathology and are not adequately controlled with dopamine replacement therapies.^2^, ^4^ Indeed, in the levodopa era, these non‐DA features represent the major source of disability for PD patients and the major reason for institutionalization. Therapy to slow or stop disease progression and to prevent the development of cumulative disability is the major unmet medical need for the treatment of PD, but to date no treatment has been approved for this indication, leaving the precise cause of neurodegeneration in PD in doubt. In this review, we will consider the mounting evidence that the cellular Abelson tyrosine kinase (c‐Abl) plays a key role in the etiopathogenesis of PD and that inhibition of c‐Abl has the potential to be a disease‐modifying therapy for PD.

## c‐Abl

**FIG 1:**
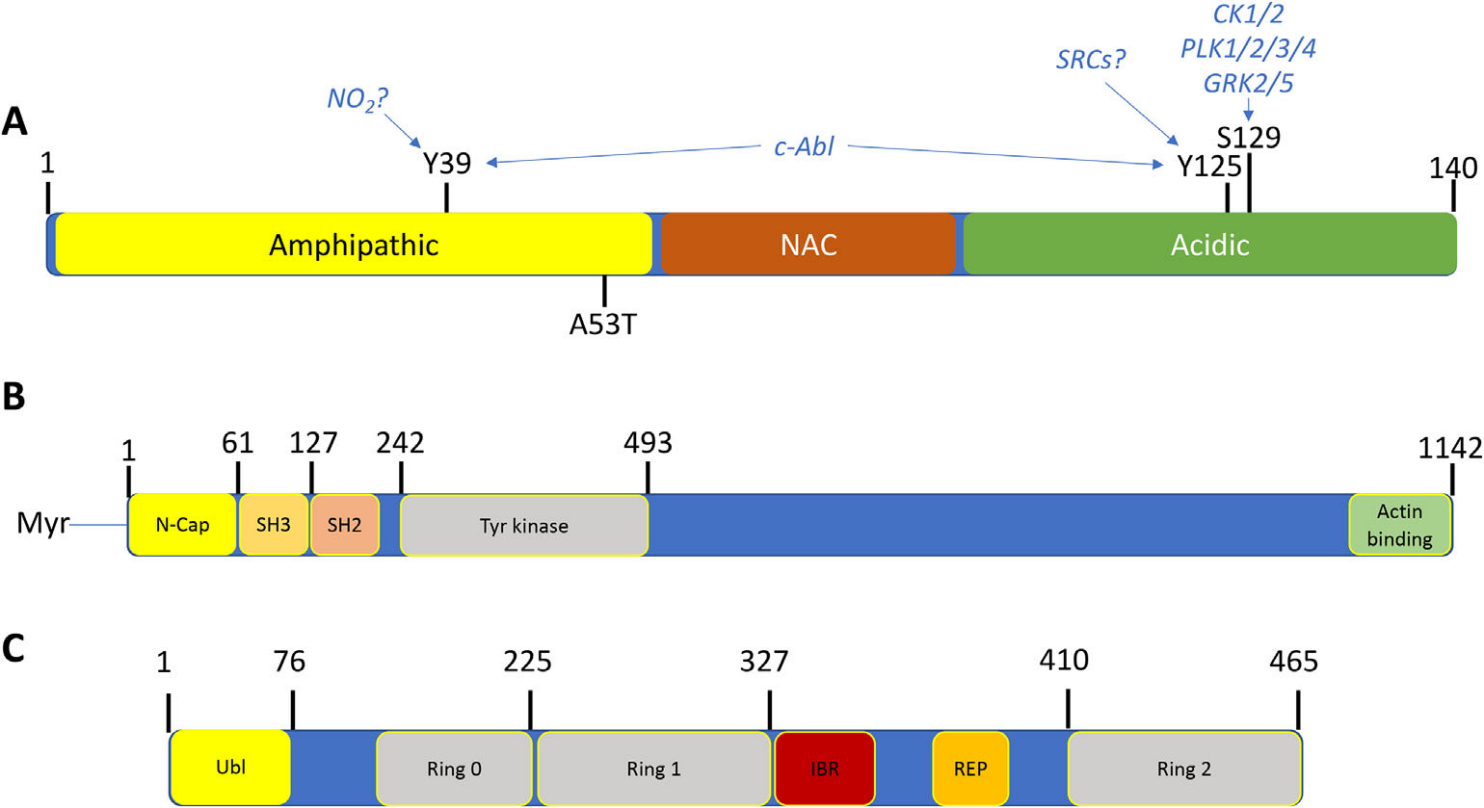
Feature map of α‐synuclein, c‐Abl, and parkin. (**A**) α‐Synuclein is a small, irregularly structured protein of 140 amino acids, which is comprised of a 61 amino acid amphipathic helical bundle where both the c‐Abl phosphorylation site at Tyr^39^ (Y39) and one of six disease associated point mutants (A53T) is located and associated with inherited Parkinson's disease. The non‐amyloid‐β‐component (NAC) is followed by an irregularly structured acidic region where both Tyr^125^ and Ser^129^ reside, whereas Tyr^39^ resides in the amphipathic coiled‐coil region. Above these chemical modification sites are the putative kinases implicated in phosphorylation of these sites. (**B**) c‐Abl is a large, multidomain protein of 1142 amino acids, wherein the regulatory regions of the myristylation cap, SH3, and SH2 domains reside in the N‐terminal half of the protein, followed by tyrosine kinase domain. The kinase core of the c‐Abl protein has a domain organization similar to that of the Src family kinases, with sequential Src homology (SH) 3 and SH2 domains, an SH2/kinase linker, and a bi‐lobed kinase domain. This core is flanked by an N‐terminal “cap” (N‐cap) region with a signal sequence for myristylation, which serves dual roles in regulation of kinase activity and in membrane localization. C‐terminal to the kinase domain is a long region of >600 amino acids encoded by a single exon, which controls interaction of Abl with other SH3‐containing proteins and the actin cytoskeleton. This region also regulates nuclear‐cytoplasmic shuttling of the kinase. (**C**) Parkin is a protein of 465 amino acids consisting of a Ubiquitin‐like domain (Ubl) at its N terminus and four zinc‐coordinating RING‐like domains: RING0, RING1, in‐between RING (IBR) and RING2, and a repressor element of parkin (REP) domain. It is classified as a RING‐between‐RING or RBR E3 ubiquitin ligase. More than 120 pathogenic PD mutations are spread throughout parkin domains, attesting to critical functions for each of the individual domain. The Ubl domain is involved in substrate recognition, binding SH3 and ubiquitin interacting motif (UIM) domains, proteasome association, and regulation of cellular parkin levels and activity.

**FIG 2:**
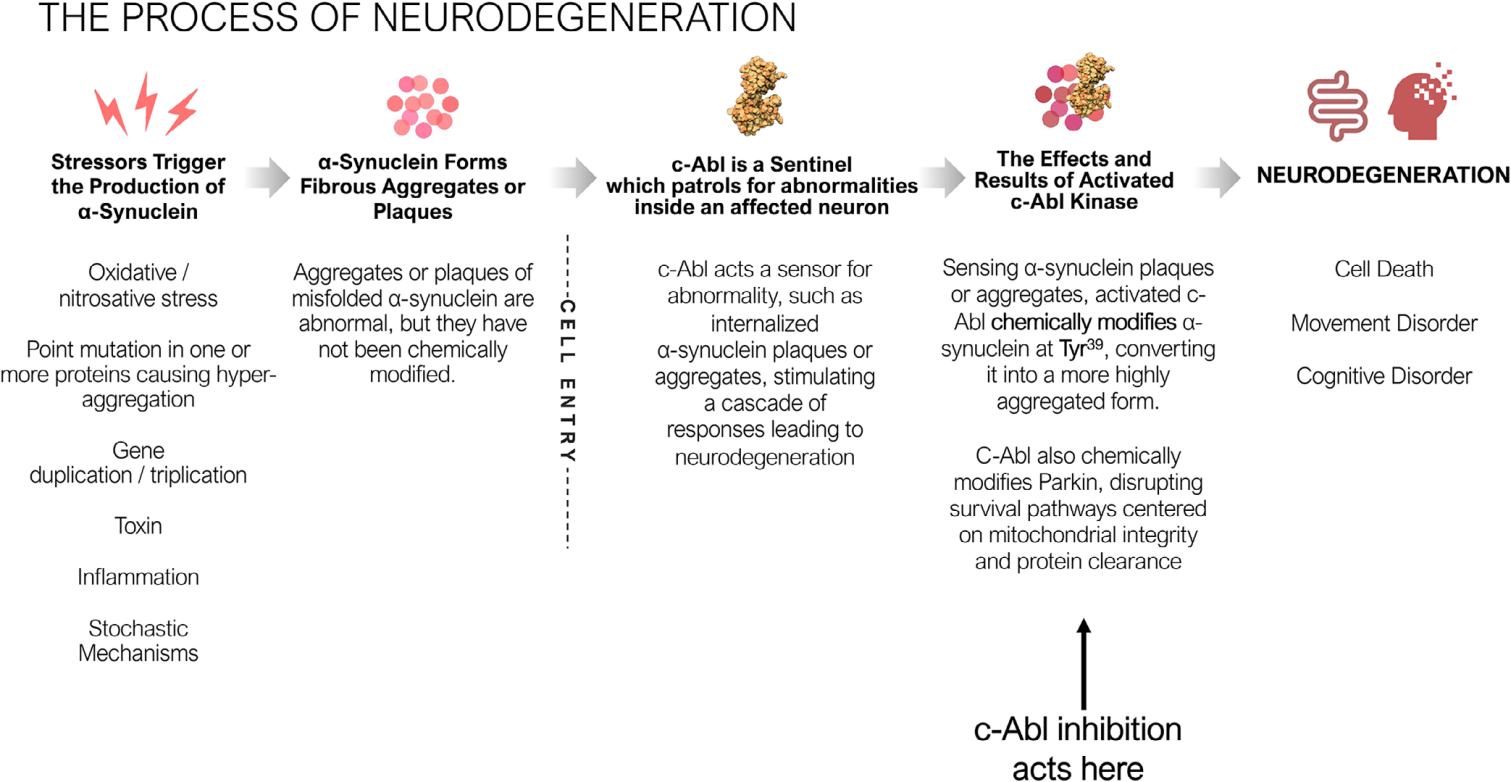
The Process of Neurodegeneration. Environmental, genetic and biochemical triggers lead to α‐synuclein aggregate formation. These aggregates are abnormal, may have diverse quaternary structures and are believed to be benign. The α‐synuclein aggregates or plaques that matter arise after the affected neurons take up the benign aggregates or plaques. Once internalized, c‐Abl, a sentinel capable of recognizing abnormalities, is activated, leading to chemical modification by phosphorylation of the internalized aggregates or plaques at Tyr39. c‐Abl also inactivates the ubiquitin E3 ligase Parkin, which drives neurodegeneration through a combination of mitophagy and/or parthanatos and shuts off pathways for clearance of phosphorylated and/or internalized α‐synuclein aggregates or plaques.

c‐Abl is a non‐receptor tyrosine kinase that is an essential sensor of cellular stresses. It acts as a sentinel to recognize abnormalities in neurons and initiates biochemical cascades driving neurodegeneration (Figs. 1 and 2).^5^ c‐Abl was first identified as the mammalian homolog of the oncogenic gene product of the Abelson murine leukemia virus.^6^ The *ABL* gene is found in all metazoans and expresses a protein of 1142 amino acids (Fig. 1). The N‐terminal Src‐homology domain 3 (SH3) and Src‐homology domain 2 (SH2) play key regulatory roles in the signaling processes mediated by the kinase domain along with the C‐terminal actin‐binding domain (ABD). These domains are conserved in the vertebrate and invertebrate *ABL* genes. Vertebrate genomes also contain a closely related isoform of *ABL*, the Abl‐related gene, or *ARG* gene.^7^ The *ABL* (*Abl1*) and *ARG* (*Abl2*) genes of vertebrates are highly conserved in the N‐terminal kinase domain and the C‐terminal ABD, but less well conserved outside of these regions. The ABD specifies localization to F‐actin, whereas the large C‐terminal region of the mammalian ABL protein includes three independently functional nuclear localization signals (NLS) that drive the nuclear entry of ABL and three cooperatively functional High Mobility Group (HMG)‐like boxes (HLB) that specify interaction with A/T‐rich DNA.^8^ The nuclear accumulation of ABL is stimulated on DNA damage.^9^, ^10^ The mammalian ABL protein undergoes nucleocytoplasmic shuttling because it is also actively exported out of the nucleus.^11^ The nuclear export of ABL requires its nuclear export signal (NES), which binds to exportin‐1 and is embedded in the ABD.^11^ *ABL* regulates many cellular processes as a consequence of this complex architecture, including the actin cytoskeleton,^12^, ^13^ the cell cycle,^14^, ^15^ and the apoptotic/cell‐cycle arrest response to stress.^5^, ^6^, ^7^, ^16^, ^17^, ^18^, ^19^, ^20^

The activities of the endogenous Abl kinases are highly regulated by diverse stimuli that range from growth factors, chemokines, DNA damage, oxidative stress, and adhesion receptors to microbial pathogens.^21^, ^22^ Once activated, the Abl kinases regulate signaling pathways implicated in cytoskeletal reorganization that are important for cellular protrusions, cell migration, morphogenesis, adhesion, endocytosis, and phagocytosis.^21^, ^23^ Abl kinases can also regulate cell survival and proliferation pathways depending on the cellular context.^24^ Emerging data support a role for abnormally activated Abl kinases in diverse pathologies, including several solid tumors, inflammatory disorders, and neurodegenerative diseases. Moreover, accumulating reports have revealed that Abl family kinase function is subverted by numerous microbial pathogens to achieve entry, motility, release, and/or survival in mammalian host cells.^24^ Therefore, targeting the Abl kinases with small‐molecule inhibitors, which were initially developed to treat patients with hematological or gastrointestinal tumors, might be used to treat distinct pathologies where Abl activation is a key driver of disease.

c‐Abl is widely expressed in the brain and plays a crucial role in neuronal development. During neurite outgrowth, c‐Abl forms a complex with and activates cyclin dependent kinase 5 (cdk5) to regulate neuronal migration and neurite outgrowth.^25^ c‐Abl also interacts directly with TrkA, N‐methyl‐D‐aspartate (NMDA), and EphB receptors, implicating c‐Abl within multiple signaling pathways of the nervous system. c‐Abl is relatively quiescent in healthy adult neurons, but c‐Abl activation occurs in the context of neurodegeneration,^26^, ^27^, ^28^ and activated c‐Abl is markedly increased in models of PD and in brain samples derived from PD patients.^28^, ^29^, ^30^, ^31^, ^32^, ^33^, ^34^ These observations led to an investigation into the role c‐Abl might play in the pathophysiology of neurodegeneration in PD and the potential therapeutic benefit of c‐Abl inhibition to treat PD.

## c‐Abl and α‐Synuclein

Much of our current thinking on the origin(s) of PD and the potential for development of a disease modifying therapy for PD has focused on α‐synuclein, and multiple therapies are currently being investigated that are designed to prevent its misfolding and/or its accumulation.^35^ Mutations in α‐synuclein cause rare cases of familial PD, and include A30P, E46K, H50Q, G51D, and A53T.^36^ Familial PD is also associated with duplication and triplication of wild‐type α‐synuclein, indicating that overexpression of the wild‐type protein alone is sufficient to cause the disease.^37^, ^38^, ^39^ Misfolded and aggregated forms of α‐synuclein are also known to be a major component of LBs,^40^ suggesting that alterations in α‐synuclein are a contributing factor to neurodegeneration in sporadic as well as familial forms of PD. The finding that Lewy pathology developed in embryonic DA neurons following transplantation into PD patients^41^, ^42^ raised the possibility that α‐synuclein could spread from affected to unaffected neurons and that PD might be a prion disorder.^43^ Subsequent studies in both transgenic and wild‐type rodents demonstrated that striatal injection of purified α‐synuclein fibrils (PFFs) or targeted expression of α‐synuclein in the striatum leads to Lewy pathology, degeneration of nigral neurons, behavioral abnormalities, and spread to neighboring regions.^40^, ^44^, ^45^ Similar findings were made in rodents and non‐human primates following injection of homogenates derived from the LBs of patients with PD or multiple system atrophy (MSA).^46^, ^47^ Importantly, these pathologic and behavioral findings were not observed following PFF injection into α‐synuclein null animals^32^, ^44^ suggesting that the process of neurodegeneration is dependent on the presence of host α‐synuclein with permissive templating, in which the accumulation of pathological α‐synuclein promotes conversion of wild‐type α‐synuclein into a pathological form in a prion‐like chain reaction.^48^, ^49^ However, despite this body of information implicating α‐synuclein in the etiopathogenesis of PD, our understanding of the precise mechanism whereby α‐synuclein causes neurodegeneration and the specific form of α‐synuclein that is toxic is not yet fully understood.

**FIG 3:**
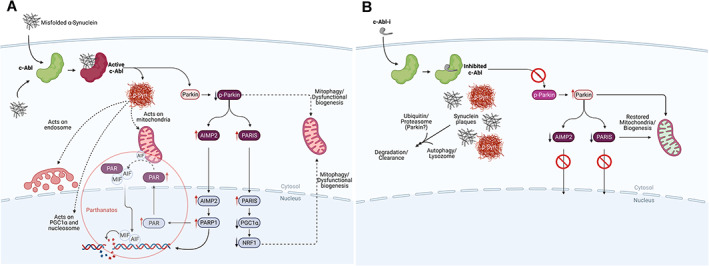
The biochemistry of Parkinson's disease initiation and progression and how to disrupt it. (**A**) The process of neurodegeneration. Misfolded α‐synuclein can arise from a variety of factors (see text). Misfolded α‐synuclein may form within the neuron or by transfer through cell surface receptors or by crossing membrane bilayers. Within a neuron, misfolded α‐synuclein is ‘sensed’ and c‐Abl activated, driving the formation of pathologic α‐synuclein by chemical modification (p‐Syn).^28^ Chemical modification creates a form of α‐synuclein that represents the pathologic species of the disease leading to disruption of mitochondrial integrity, negatively impact the endosome, disrupt nucleosomal structure and modulate transcription of certain genes.^32^, ^33^, ^34^, ^35^, ^36^, ^37^, ^38^, ^63^, ^64^, ^65^, ^66^, ^67^, ^68^, ^69^, ^70^, ^71^, ^72^ C‐Abl also inactivates parkin by chemical modification, which affects mitochondrial quality control and suppresses protein clearance mechanisms.^28^, ^29^, ^30^, ^31^, ^32^, ^33^, ^34^, ^77^, ^78^, ^79^ Parkin inactivation suppresses the complex interplay between parkin and pink1 at the mitochondrion, which act in concert to maintain mitochondrial integrity, quality and regulate mitochondrial biogenesis.^77^ Parkin inactivation leads to the accumulation of toxic parkin substrates, such as the parkin interacting substrate (PARIS), the aminoacyl tRNA synthetase complex‐interacting multifunctional protein 2 (AIMP2) and the far upstream element‐binding protein 1 (FBP1).^28^, ^29^, ^30^, ^31^, ^32^, ^33^, ^34^, ^77^, ^78^, ^79^ PARIS and AIMP2 accumulate in adult conditional parkin knockout mice and MPTP‐intoxicated mice as well as in patients with PD. Increased levels of PARIS can lead to mitochondrial dysfunction through downregulation of the peroxisome proliferator‐activated receptor‐gamma coactivator (PGC)‐1α transcriptional co‐activator protein and loss of DA neurons in a PARIS‐dependent manner. PGC1‐α is a transcriptional co‐activator that plays a central role in regulation of energy metabolism. Overexpression of AIMP2 leads to an age dependent, selective degeneration of DA neurons through activation of poly (ADP‐ribose) polymerase 1 (PARP1), driving PARP1‐mediated parthanatos. This suggests that PARIS and AIMP2 may be important contributors to the loss of DA neurons and possibly other vulnerable neurons following parkin inactivation. Inactivation of parkin also disrupts protein clearance mechanisms through autophagy, lysosomal, and proteasomal degradation pathways. (**B**) The consequences of c‐Abl inhibitor treatment on the process of neurodegenerative disease. Inhibition of c‐Abl precludes c‐Abl activation, blocking the build‐up of toxic parkin substrates PARIS and AIMP2 and terminating downstream events. This also re‐establishes normal mitochondrial quality control and biogenesis. Model studies demonstrate that modified and unmodified α‐synuclein aggregates are shunted to lysosomal or proteasomal degradation pathways for clearance with concomitant recovery of motor function.

Recent studies have begun to shed light on the biochemical pathways that underlie how pathologic α‐synuclein might stimulate cell death and suggest that the cellular c‐Abl plays a key role (Figs. 2 and 3). α‐Synuclein is subject to a wide range of post‐translational modifications, which include acetylation, glycation, glycosylation, nitration, sumoylation, ubiquitination, cross‐linking, truncation, and phosphorylation (Fig. 1).^50^ Much attention has focused on phosphorylation of α‐synuclein at Ser^129^ (pS129) because antibodies to pS129 are the standard way of visualizing Lewy pathology. Under normal conditions, only a small fraction of α‐synuclein is constitutively phosphorylated at Ser^129^ in human brain.^51^, ^52^, ^53^ However, there is a dramatic accumulation of pS129 α‐synuclein in transgenic animal models and in the brains of patients suffering from PD.^53^, ^54^, ^55^, ^56^ These findings led to the hypothesis that pS129 is the pathologic or toxic form of the protein. However, pS129 is not required for the formation of LB‐like inclusions or neurodegeneration that occurs following PFF injection into wild‐type mice.^32^ Mutation of Ser^129^ to Ala (S129A) in rodent brain, which prevents pS129 formation, does not prevent α‐synuclein misfolding nor the development of PD‐like pathology.^32^, ^57^, ^58^ Therefore, the absence of a serine phosphorylation site does not prevent the development of pathologic or behavioral abnormalities in animal models, raising questions as to the importance of pS129 in the PD process. The discovery of alternatively phosphorylated forms of α‐synuclein, and particularly phosphorylation at Tyr^39^ (pY39)^28^ and Tyr^125^ (pY125)^59^ by c‐Abl, raised the possibility that c‐Abl could be a key player in the neurodegenerative disease process. Phosphorylation at the Tyr^125^ site does not interfere with other post‐translational modifications or alter fibril formation in vitro and does not appear to be an essential part of α‐synuclein pathology.^60^ Phosphorylation at Tyr^39^, on the other hand, is directly linked to the development and progression of α‐synuclein pathology in multiple mouse models^28^, ^31^, ^61^, ^62^ and has been observed in post‐mortem brain samples from PD patients but not in age‐matched controls.^28^

To determine if pY39 is a marker for the critical pathologic form of α‐synuclein, it needed to be shown that activation of c‐Abl is a requirement for neurodegeneration to occur and that inhibition of the enzyme prevents neurodegeneration. There is now considerable evidence supporting both of these hypotheses. Expression of a constitutively active form of c‐Abl accelerates neurodegeneration in transgenic mice expressing the alanine‐to‐threonine mutation at position 53 of α‐synuclein (hA53T).^28^ By contrast, when c‐Abl is genetically deleted in these models, neurodegeneration does not occur.^31^ Similarly, in animal models of PD, pharmacological inhibitors of c‐Abl reduce the formation of pY39 α‐synuclein and Lewy pathology, reduce mitochondrial dysfunction, and rescue DA neurons and motor function in a dose‐dependent manner. These findings highlight the fundamental importance of c‐Abl activation to the disease process.^63^, ^64^, ^65^, ^66^ Indeed, several c‐Abl inhibitors including imatinib,^29^ nilotinib,^61^, ^67^, ^68^ bafetinib,^69^ radotinib,^61^ and the novel inhibitors in clinical development vodobatinib/SCC‐138/K0706^70^ and IkT‐148009^71^, ^72^ have all demonstrated that c‐Abl inhibition can act prophylactically and/or therapeutically to counter neurodegeneration in animal models of PD.

Based on the observations that c‐Abl knockout^28^ or chemical inhibition^28^, ^61^, ^67^, ^68^, ^69^ is neuroprotective in animal models of PD, we believe that c‐Abl activation may be a key driver of neurodegeneration. Indeed, activated c‐Abl is markedly increased in models of PD and in brain samples derived from PD patients.^28^, ^62^, ^73^ These data suggest that c‐Abl activation followed by formation of pY39 may be a required step for neurodegeneration to occur, whereas phosphorylation at Ser^129^ may just be a marker of misfolded α‐synuclein, but not a modification critical to the neurodegenerative disease process itself. The failure of poorly brain penetrant c‐Abl inhibitors like nilotinib to show a consistent neuroprotective phenotype in model studies^62^ or clinical trials (see below), is likely to be a result of incomplete inhibition of c‐Abl activation, not a sign that c‐Abl is insignificant to the disease process.

## c‐Abl and Parkin

There is also evidence that activation of c‐Abl promotes neuronal degradation by way of its engagement with parkin, in addition to its effects on α‐synuclein (Figs. 1 and 3). Parkin is a ubiquitin E3‐ligase that promotes ubiquitination and clearance of unwanted proteins via the ubiquitin‐proteasome pathway.^74^ It also acts in concert with pink1 to monitor and preserve mitochondrial integrity.^29^, ^30^, ^75^, ^76^, ^77^ In response to oxidative or nitrosative stress, c‐Abl is activated and phosphorylates parkin at Tyr^143^ (pY143‐parkin), resulting in the loss of its enzymatic activity.^29^, ^30^ This results in impairment of protein clearance pathways dependent on parkin activity (Figs. 2 and 3).^30^, ^31^ Inactivation of parkin also suppresses the complex interplay between parkin and pink1, stimulating mitophagy and other downstream events that ultimately result in the degradation of affected neurons (see Fig. 3).^77^, ^78^, ^79^ Parkin inactivation by c‐Abl further triggers a signaling cascade that leads to poly‐ADP‐ribose polymerase‐1 (PARP‐1)‐mediated cell death or parthanatos, a form of cell death that that involves alterations in DNA repair and DNA transcription with resultant mitophagy (see Fig. 3).^31^, ^33^, ^34^, ^77^, ^78^, ^79^

The relevance of parkin to the Parkinson's disease process was first appreciated when mutations in parkin were discovered to cause an autosomal recessive, young‐onset form of PD in Japanese families.^80^ Further, DA neurodegeneration with locomotor deficits is observed in parkin knock‐out mice.^78^, ^81^ Treatment with a c‐Abl inhibitor, or genetic deletion of c‐Abl, suppresses Tyr^143^ phosphorylation of parkin, promotes protein clearance and protects against neurodegeneration.^29^, ^31^, ^61^, ^67^, ^68^, ^69^ Specifically, the c‐Abl inhibitors bafetinib (aka INNO‐406), radotinib and nilotinib block inactivation of parkin by c‐Abl and protect against neurotoxicity in mouse models of PD.^61^, ^67^, ^68^, ^69^ Similarly, in conditional c‐Abl knockout mice, tyrosine phosphorylation of parkin and neurodegeneration do not occur.^31^ These observations link c‐Abl activation and phosphorylation of parkin to the disruption of survival pathways that ordinarily protect neurons from the adverse effects of a toxin or accumulation of deleterious proteins like misfolded α‐synuclein.

## Inhibition of c‐Abl as a Potential Disease‐Modifying Therapy for PD

Considering the evidence suggesting that c‐Abl activation might play a key role in the etiopathogenesis of PD, it is rational to consider that inhibition of c‐Abl activation could be a disease‐modifying therapy. Clinically, c‐Abl inhibitors have been in use for more than 20 years, primarily as a treatment for patients with hematological and gastrointestinal cancers. Imatinib mesylate, marketed as Gleevec®, is the standard‐of‐care treatment for Philadelphia chromosome‐positive chronic myelogenous leukemia. Nilotinib, marketed as Tasigna®, followed imatinib into the clinic and was developed for patients who develop resistance or intolerance to imatinib. Both drugs are associated with significant hematological adverse events (AEs) (neutropenia, leukopenia, and cytopenia) in patients with chronic myelogenous leukemia; other frequently observed adverse effects include periorbital edema, anemia, and chronic gastrointestinal (GI) disturbances. Nilotinib also carries a black box warning related to cardiovascular risks, particularly QTcF prolongation. Importantly, the hematological AEs associated with kinase inhibitor therapy in patients with cancer have not been observed in non‐oncology patients.^81^, ^82^, ^83^, ^84^, ^85^

Several clinical trials have tested nilotinib in PD patients. Nilotinib was initially studied in a 6‐month, open label trial evaluating 150 or 300 mg in 12 patients with PD dementia/dementia with LBs.^86^ Unified Parkinson's Disease Rating Scale (UPDRS) motor scores were reported to have improved by ~3.5 points at 24 weeks, but benefits were not seen at 36 weeks. Interestingly, patients reported an increased frequency of bowel movements in this study, which could reflect a benefit on this non‐motor feature of PD. One patient suffered a myocardial infarction and two had transient QTcF prolongation. Subsequently, two placebo‐controlled, double‐blind trials tested Nilotinib in patients with moderately advanced PD.^87^, ^88^, ^89^ The first was a 12‐month single center study in 75 patients who were randomly assigned 1:1:1 to Nilotinib (150 or 300 mg) or placebo.^87^ Nilotinib had no effect on motor function during the treatment phase or during a 3‐month post‐treatment follow‐up period, but patients were also receiving concurrent anti‐parkinsonian medications, which could have confounded results. Serious adverse events (SAEs) were noted in 13 Nilotinib‐treated patients and included three cardiovascular events; the other SAEs were not considered to be treatment‐related. Sixty‐three of these patients participated in a 12‐month open‐label extension study; no meaningful improvement in clinical features of PD as measured by the UPDRS, Parkinson's Disease Questionnaire (PDQ‐39) and Montreal Cognitive Assessment (MoCA) assessments was detected.^88^ In a second study, 76 PD patients participated in a 25 center, 6‐month double‐blind trial and were randomized 1:1:1 to receive placebo or Nilotinib (150 or 300 mg).^89^ The treatment was generally well tolerated, with the most frequent AE being an increase in amylase levels, as has been reported with other kinase inhibitors.^90^ However, no meaningful clinical benefit was observed as measured by the MDS‐UPDRS total score in either the ON or OFF state; indeed, there was a trend toward worsening in comparison to the placebo group.^89^ Further, there were no nominally significant benefits for any other clinical outcome measure and no meaningful effect on biomarkers was detected. Therefore, double‐blind studies performed to date have not replicated the benefits reported in the open label study.

The failure of Nilotinib to provide a clinically meaningful benefit in PD patients in two double‐blind studies is discouraging, but may be explained by the fact that nilotinib does not accumulate in the brain at concentrations sufficient to inhibit c‐Abl.^91^ As a competitive inhibitor of c‐Abl with an IC_50_ of ≈48 nM, Nilotinib would require a sustained concentration of 150 nM in the brain for inhibition of the enzyme. Nilotinib does not appear to have reached this threshold as cerebrospinal fluid (CSF) measures of Nilotinib reached only a maximum of 10% of the concentration thought to be required for adequate inhibition of c‐Abl,^91^ and even that level was not sustained throughout the day. This may be because of the fact that Nilotinib, and with other c‐Abl inhibitors developed as anticancer agents, are substrates for efflux transporters like ABCG2 (aka P‐glycoprotein or PGP) and ABCB1 (aka MDR1 or C8), which promote their removal from the brain and, therefore, make it difficult to achieve saturating concentrations in the brain to inhibit c‐Abl.

At least two novel c‐Abl inhibitors (K0706/SCC‐138/Vodobatinib^70^ from Sun Pharma Advanced Research Company [SPARC] and IkT‐148009^71^, ^72^ from Inhibikase Therapeutics) are currently in clinical development that may have overcome the barrier to achieving adequate brain concentrations, and in animal studies, have been reported to accumulate in the brain at levels sufficient to inhibit c‐Abl^70^, ^71^, ^72^ Vodobatinib is essentially a chemical amalgam of the commercial anticancer agents Dasatinib and Ponatinib in which the ATP‐binding domain of Ponatinib is linked to the active site binding domain of Dasatinib.^92^ It has a reported IC_50_ for wild‐type c‐Abl of 0.9 nM, comparable to Ponatinib. By contrast, IkT‐148009 is a chemical derivative of the commercial anticancer agent Imatinib, in which in the active site binding domain remains unchanged, but the ATP‐binding site domain has been chemically altered to enhance the inhibitory potency of IkT‐148009 for wild‐type c‐Abl. These changes result in an IC_50_ of 33 nM for c‐Abl, an improvement in potency of more than 20‐fold over Imatinib.

Insight into potential efficacy of these agents has not been published in peer reviewed journals, but has been reported in a non‐peer review format.^70^, ^71^, ^72^ Vodobatinib was reported to have been tested in both PFF and hA53T rodent models of PD and to show a dose‐dependent preservation of DA neurons with functional improvement in both the pole and limb force tests.^70^ IkT‐148009 was evaluated in several different models, including 1‐methyl‐4‐phenyl‐1,2,3,6‐tetrahydropyridine (MPTP)‐treated mice, transgenic and inducible hA53T rodent models, and in the PFF rodent model.^71^ In each of the hA53T and PFF models, IkT‐148009 cleared α‐synuclein aggregates from the brain and GI tract, prevented the loss of nigrostriatal neurons, promoted regeneration of enteric neurons, and induced substantial functional recovery in a battery of measures of motor and non‐motor functions.^71^, ^72^ IkT‐148009 was also evaluated in the presence and absence of elacridar, a ABCG2/PGP inhibitor. Elacridar provided no added neuroprotective benefit by blocking the efflux transporter PGP, suggesting that IkT‐148009 was not affected by the efflux transporter and had reached saturating values at steady state in the brain.

Clinical trials have now been initiated with both agents (NCT03316820, NCT02629692, NCT03655236, and NCT04350177). In an open‐label phase 1 dose escalation trial in patients with chronic myelogenous leukemia (N = 35), Vodobatinib was generally well tolerated in doses up to ≈200 mg, but reached dose‐limiting toxicities at 240 mg.^93^ The most common AEs were GI disturbances (18.5%), myalgia and fatigue (15.7%), and neutropenia and thrombocytopenia (12.7%). Other AEs included dyspnea and non‐cardiac chest pain, both of which returned to normal following dose‐reduction.^93^ In a 14‐day placebo‐controlled trial in 46 PD patients, Vodobatinib was reported to be well‐tolerated with no serious clinical or laboratory AEs that were considered to be drug‐related. Plasma concentrations are reported to have reached levels that were efficacious in PD animal models and CSF measures.^94^ IkT‐148009 was evaluated in a randomized phase 1 dose‐escalation study at doses up to 100 mg in older healthy adults, ages 45 to 70 (N = 56), with no clinically meaningful AEs. Laboratory abnormalities noted a mild and transient increase in amylase and/or lipase, common to drugs in this class.^90^ No hematological or GI AEs have been observed following daily dosing up to 7 days. Adequately powered placebo‐controlled studies testing the effects of these agents on measures of disease progression and the development of cumulative disability are planned or ongoing.

## Conclusions

Advances in our understanding of the processes leading to neurodegeneration in relevant animal models of PD have led to an alternative view of the early steps that govern the initiation and progression of neurodegeneration. These studies argue that activation of c‐Abl plays a critical role in the formation of pathological α‐synuclein species, inhibition of the protective effects provided by parkin, and in the evolution of the neurodegenerative disease process in PD. Studies further indicate that inhibition of c‐Abl prevents these changes in animal models of PD. Collectively, this evidence suggests that c‐Abl activation, and not just α‐synuclein accumulation, is driving the downstream effectors of neurodegeneration in PD. We hypothesize that the misfolded α‐synuclein that matters in PD forms inside the affected neurons following activation of c‐Abl and that activated c‐Abl creates the toxic form of α‐synuclein containing pY39 (and possibly additional modifications) that initiates the neurodegenerative disease process. Importantly, if the neurodegenerative disease process is dependent on activation of c‐Abl, with generation of pathologic forms of α‐synuclein through tyrosine phosphorylation, then the critical driver of the disease process resides inside the affected neurons. This may explain why immunotherapy approaches studied to date in PD patients have failed in clinical trials, as antibodies cannot gain access to the pathologic form of α‐synuclein.^95^, ^96^

Viewed from this perspective, we believe that inhibition of c‐Abl activation could have disease modifying effects that could slow or halt disease progression in PD. Double‐blind clinical trials of the c‐Abl inhibitor Nilotinib failed to show clinical benefits in PD patients. Although Nilotinib is a potent inhibitor of c‐Abl peripherally, it has relatively poor brain penetration (brain:plasma ratio <0.1% in mice).^61^, ^67^ Accordingly, Nilotinib had inconsistent and only modest neuroprotective effects in mice following oral administration and has not been shown to eliminate the accumulation of phosphorylated synuclein aggregates from the brain in synuclein‐dependent progressive disease models.^61^ We believe that the failure of nilotinib to provide clinical benefits in PD patients is likely to be a result of incomplete inhibition of c‐Abl activation, and not a sign that c‐Abl is insignificant to the disease process. Novel c‐Abl inhibitors have been developed that may not bind to efflux transporters and, therefore, have the potential to provide greater brain concentrations and saturating levels of c‐Abl inhibition, show promising benefits in preclinical models of PD, and have demonstrated good safety profiles in early clinical studies albeit in a limited number of PD patients.

Although there is no assurance that the positive findings surrounding c‐Abl inhibition in animal models will translate into clinical benefits in PD patients, we believe, based on available data, it is important to test these new molecules in well controlled double‐blind trials to establish whether the promise of mechanistic understanding in models translates into meaningful benefits for PD patients. The patient population for testing these agents in clinical trials is generally considered to be early, untreated, PD patients. This avoids the confounding effects of symptomatic medications, which might interfere with interpretation of study results. Further, in this population there is a greater likelihood of surviving DA neurons as anatomic studies suggest that DA neurons have largely degenerated by as little as 4 years after diagnosis.^97^ Enrichment strategies will be sought, but initially there are no validated biomarkers or clinical syndromes that would permit determining which patient population is most likely to respond to c‐Abl inhibitors—indeed, we believe that these therapies should be beneficial for all PD patients. The delayed‐start design is the only approach that regulatory authorities have indicated would permit approval with an indication that the treatment is disease‐modifying. However, we prefer a more rapid and efficient development approach that would use a standard parallel group, placebo‐controlled, double‐blind study that includes endpoints that measure the rate of UPDRS progression and features such as gait impairment, cognitive dysfunction, and cumulative disability that are not meaningfully affected by currently available therapies.^98^ Although this approach would not likely result in a label indication for disease‐modification, a description of the relevant basic science studies and clinical endpoints could be included in sections XII and XIV of the label and could, therefore, be used for educational and promotional purposes.^98^

## Financial Disclosures

M.H.W. is an employee of and the largest shareholder in Inhibikase Therapeutics. C.W.O. owns stock in Clintrex Research Corporation, which provides medical consulting services to Inhibikase Therapeutics and C.W.O. owns stock options in Inhibikase Therapeutics. C.W.O. is Interim Chief Medical Officer of Inhibikase Therapeutics through a consulting contract between Inhibikase and Clintrex.

## Author Roles

(1) Research project: A. Conception, B. Organization, C. Execution; (2) Statistical Analysis: A. Design, B. Execution, C. Review and Critique; (3) Manuscript: A. Writing of the First Draft, B. Review and Critique.

M.H.W.: (1 A, B, C), (3 A, B)

C.W.O.: (1 B, C), (3 B)

## Data Availability

Publicaly available data is evaluated in this manuscript
